# Double-stacked hyperbolic metamaterial waveguide arrays for efficient and broadband terahertz quarter-wave plates

**DOI:** 10.1038/s41598-017-00726-3

**Published:** 2017-04-03

**Authors:** Xianmin Ke, Hua Zhu, Junhao Li, Lin Chen, Xun Li

**Affiliations:** 1 Wuhan National Laboratory for Optoelectronics, Huazhong University of Science and Technology, Wuhan, 430074 China; 2 Department of Electrical and Computer Engineering, McMaster University, 1280 Main Street West, Hamilton, Ontario L8S 4L8 Canada

## Abstract

We demonstrate how it is possible to achieve weak dispersion in the phase delay between two orthogonal polarization states by using double-stacked hyperbolic metamaterial (HMM) waveguide arrays. The weak dispersion in the phase delay originates from the different signs of phase delay from the two different HMM waveguide arrays. The condition of dispersion-free phase delay for the transmitted waves has been theoretically derived from the transmission matrix as the propagation characteristic of the HMM waveguide is involved. We further reveal that the designed double-stacked HMM waveguide array can function as an efficient quarter-wave plate that enables the conversion of linearly polarized light to circularly polarized light within a broad frequency band. In addition, the bandwidth over which the degree of linear polarization is nearly unity and over which the angle of linear polarization is kept at approximately 45° is basically consistent with the phase bandwidth. This offers a promising approach for developing a practical polarization converter in the terahertz domain.

## Introduction

The ability to manipulate the polarization states of light is of central importance for many applications in physics, chemistry, biology, and optics^1^. Conventionally, a birefringent crystal plate with a certain thickness, known as a “wave plate,” is employed to manipulate the polarization states of light. The phase difference between two orthogonal directions of light depends on the birefringence and the thickness of the crystal plate. However, it is inconvenient to integrate birefringent crystals with other optical components in optical circuits due to their bulky volume, which originates from their small birefringence index (typically, less than 0.2).

As one of the least-explored areas in the electromagnetic (EM) spectrum, terahertz (THz) science and technology exhibits great potential in the fields of communication, imaging, biological diagnosis, and chemical sensing^2–4^. It is highly anticipated that these applications may significantly benefit from polarization management, since the polarization state is one of the key characteristics of EM waves. However, most natural materials present a weak response to THz waves, making the development of THz polarization manipulation components significantly inferior to their counterparts in the optical domain^5^. Therefore, exploration of new mechanisms targeted towards manipulation of the polarization states of EM waves will be valuable.

Emerging within the past two decades, the fields of plasmonics and metamaterials have offered many exciting methods for exhibiting a variety of exotic optical phenomena and material properties that are unattainable with conventional materials^6, 7^. Numerous metallic structures have been proposed to control the polarization states of transmitted or reflected light^8–27^. High-efficiency and broadband linear polarization converters based on metamaterials have been realized for both transmission and reflection modes in gigahertz^21, 22^, terahertz^13, 27^, infrared^19, 25^, and optical frequency regimes^16, 23^. Recently, a great deal of effort has been devoted to developing high-efficiency and broadband linear-circular polarization conversion in the reflection and transmission modes by exploiting gap-plasmon resonators^14^, plasmonic metasurfaces^11, 12, 26^, plasmonic nanorods^17, 20^ and multilayer metamaterials^18, 24^. However, from the view of practical applications, there remains the significant challenge of overcoming the strong dispersion in the transmission and reflection coefficients for different polarizations within the phase bandwidth of operation^11, 17, 24, 26^. Recent research concerning metamaterials has partially shifted to hyperbolic metamaterials (HMMs) due to their outstanding electromagnetic properties, including the strong enhancement of spontaneous emission, negative refraction and superlensing effects^28–31^. It has also been demonstrated that an HMM waveguide can be constructed for wideband photon harvesting^32^ and light guiding at the ultra-deep subwavelength scale^33^. Quite recently, we have demonstrated that an HMM waveguide array with a rectangular waveguide cross-section exhibits a giant modal birefringence index^34^, which is dozens of times higher than that of conventional quartz birefringent crystals for THz waves^35^. The birefringence index of the HMM waveguide array is comparable to or even higher than that based on various metamaterials in the THz domain^36–38^. More interestingly, the designed polarization manipulation devices with such an HMM waveguide array show the capability of converting linearly polarized light waves to circularly polarized light waves with high transmission. However, their operation bandwidth is very limited, since the HMM polarization manipulation components show strong dispersion in the phase delay between two orthogonal directions of light. Once the device operates away from the optimal wavelength, the phase delay is no longer kept at a constant value, which seriously restricts the HMM polarization manipulation components for broadband applications.

In this article, we have explored double-stacked HMM waveguide arrays to engineer the phase delay for the broadband manipulation of light polarizations. By incorporating the propagation characteristic of the HMM waveguide into the general transmission matrix, we have derived the conditions for achieving dispersion-free phase delay for transmitted waves. In cases where the cross-section parameters of the HMM waveguides are given, the phase delay can be kept constant within a broad frequency range of interest by properly arranging the heights of the HMM waveguides. Numerical simulation results demonstrate that the designed quarter-wave plates enable the conversion of linearly polarized light to circularly polarized light within a wide spectral range. In addition, the double-stacked HMM polarization manipulation components show an excellent figure of merit in terms of the degree of linear polarization (DoLP) and the angle of linear polarization (AoLP) due to the weak dispersion in the amplitude transmission for different polarizations.

## Results and Discussion

### A single-sized HMM waveguide array

**Figure 1:**
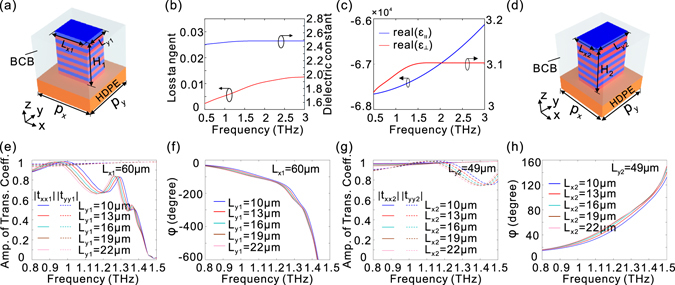
The amplitude of transmission coefficients and phase delay for HMM waveguide arrays. (**a**) Schematic of a unit cell of an HMM waveguide array with length L_x1_, width L_y1_, and height H_1_. The lattice constants along x, y, and z directions are p_x_, p_y_, and p_z_ (=t_m_ + t_d_), respectively. t_m_ and t_d_ represent the thicknesses of the metal and dielectric layers, respectively. (**b**) The loss tangent and dielectric constant of polymer BCB used in our work. (**c**) The real part of the effective dielectric permittivities (ε_||_, ε_⊥_) for the HMM considered. ε_∥_ and ε_⊥_ represent the effective dielectric permittivities perpendicular and parallel to the normal direction of the layered structure, respectively. The thicknesses of the metal and dielectric layers are set to be t_m_ = 1 μm and t_d_ = 4 μm, respectively. (**d**) Schematic of a unit cell of a HMM waveguide array with length L_y2_, width L_x2_, and height H_2_. Dependence of the amplitude of transmission coefficients |*t*_*xx*1_| (solid lines), |*t*_*yy*1_| (dashed lines) (**e**), and phase delay (**f**) on L_y1_ with L_x1_ = 60 μm, p_x_ = p_y_ = 80 μm, H_1_ = 50 μm. Dependence of the amplitude of transmission coefficients |*t*_*xx*2_| (solid lines), |t_*yy*2_| (dashed lines) (**g**), and phase delay (**h**) on L_x2_ with L_y2_ = 49 μm and H_2_ = 50 μm. The other structural parameters are the same as those in (**e**). The simulation work is conducted by numerical simulations with Lumerical FDTD solutions [see the detail in the Methods Section].

Figure 1(a) is a schematic representation of a unit cell of an HMM waveguide array with a rectangular cross-section, where a subwavelength Al/polymer benzocyclobutene (BCB) multilayer is placed on the low-loss high-density polyethylene (HDPE) substrate and the surrounding dielectric layer is BCB. The relative permittivity of the Al layers is described by the Drude model ${\varepsilon }_{m}=1-\frac{{f}_{p}^{2}}{f(f-i{f}_{\tau })}$, where *f*_*p*_ = 3570 THz and *f*_*τ*_ = 19.4 THz^39^. The polymer BCB is widely used in THz applications on account of its ultra-low loss, and its relative permittivity can be extracted from experimental data^40^ [Fig. 1(b)]. The HDPE is selected as the substrate due to its low refractive index (1.54), high stability, and small absorption coefficient in the THz region^41^. It should be noted that the HMM considered here should be classified as type II^42^, since effective medium theory^43^ indicates that the estimated effective dielectric permittivities parallel and perpendicular to the normal direction of the layered structure are positive and negative [Fig. 1(c)], respectively. Here, the TM (TE) mode of the HMM waveguide array is defined as the electric field oriented parallel to the x (y) direction. It has been demonstrated that such an HMM waveguide array exhibits a giant modal birefringence for the transmitted wave, which can be dozens of times larger than that of the birefringent crystals for THz waves^34^. Consequently, an HMM waveguide array can manipulate light polarizations with a subwavelength thickness.

We first consider an HMM waveguide array with a rectangular cross-section [L_x1_ > L_y1_, Fig. 1(a)], which leads to different cut-off frequencies for different polarizations. In this case, the cut-off frequency for the TM mode (1.48 THz) is much smaller than that for the TE mode [Fig. 1(e)]. It is well known that slow light occurs if the light frequency approaches the cut-off frequency and hence significantly enhances the photonic density of states^44^, which results in strong absorption of the EM wave^32, 45–48^. Thus, as light frequency approaches the cut-off frequency for the TM mode, the transmission amplitude of the TM mode is highly suppressed due to the slow-light effect, while that for the TE mode remains high [Fig. 1(e)]. Meanwhile, the phase delay between the TE and TM modes increases sharply near the cut-off frequency for the TM mode [Fig. 1(f)], originating from the fact that the propagation constant for the TM mode is significantly increased^34^. However, to operate as an efficient and broadband quarter-wave plate, the transmission of large amplitude and weak dispersion in the phase delay between two orthogonal directions of light should be achieved simultaneously. Despite the fact that the amplitude transmission can be kept at a high level provided light frequency is far from the cut-off frequency [Fig. 1(e)], such an HMM waveguide array suffers severely from the dispersion in the phase delay between the TE and TM modes, which significantly restricts its broadband applications [Fig. 1(f)].

**Figure 2:**
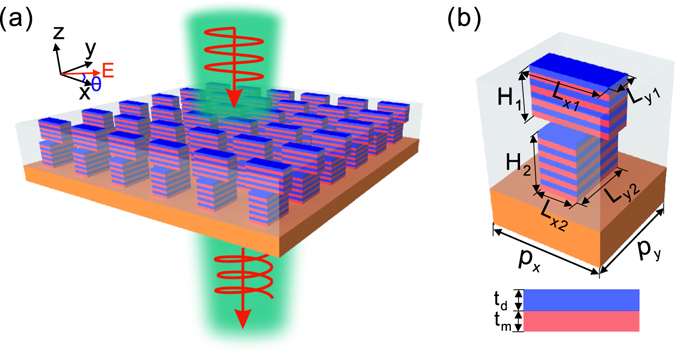
Double-stacked HMM waveguide arrays. Schematic illustrations of (**a**) double-stacked HMM waveguide arrays and (**b**) the unit cell. The HMM waveguide arrays are placed on the HDPE substrate and the surrounding material is BCB. The unit cell consists of two HMM waveguides, of which the upper (lower) one has length L_x1_ (L_y2_), width L_y1_ (L_x2_) and height H_1_ (H_2_). The lattice constants along x and y directions are p_x_ and p_y_, respectively. t_m_ and t_d_ represent the thicknesses of the metal and dielectric layers, respectively. A normal incident plane wave illuminates the structure from the –z direction with the polarization angle θ relative to the x-axis.

We next consider another HMM waveguide array with a rectangular cross-section [L_y2_ > L_x2_, Fig. 1(d)]. It is interesting to note that a comparable amplitude transmission is maintained [Fig. 1(g)], while the slope of phase dispersion changes from negative [Fig. 1(f)] to positive [Fig. 1(h)]. The opposite sign of phase dispersion indicates the possibility of compensating the phase dispersion across the chosen frequency band by combining two HMM waveguide arrays to form a double-stacked HMM waveguide array [Fig. 2]. Once the phase delay is tuned to show a flat response, the double-stacked HMM waveguide array is expected to function as a polarization manipulation component that operates within a wide spectral band.

### Broadband quarter-wave plate based on double-stacked HMM waveguide arrays

**Figure 3:**
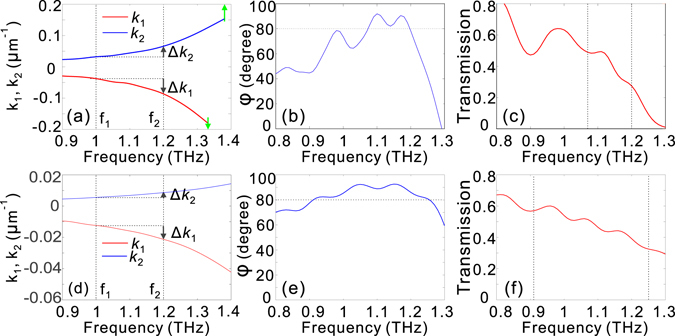
The performance of double-stacked HMM waveguide arrays with two cases of side dimensions. (**a**,**d**) *k*_1_ and *k*_2_, (**b**,**e**) phase delay, (**c**,**f**) transmission as a function of light frequency with (**a**–**c**) H_1_ = 115 μm, H_2_ = 185 μm, L_x1_ = 65 μm, L_y1_ = 20 μm, L_x2_ = 20 μm, L_y2_ = 62.5 μm, and (**d**–**f**) H_1_ = 400 μm and H_2_ = 1125 μm, L_x1_ = 50 μm, L_y1_ = 20 μm, L_x2_ = 20 μm, L_y2_ = 40 μm. In (**a**), the two vertical green arrows represent the cut-off frequencies for the TE (lower HMM waveguide) and TM (upper HMM waveguide) modes, respectively. In (**d**), the cut-off frequencies for the TE and TM modes are out of the frequency range considered. The vertical dashed lines in (**c**) and (**f**) represent the phase bandwidth of operation. The lattice constants along x and y directions are p_x_ = 80 μm and p_y_ = 80 μm, and t_m_ = 1 μm, t_d_ = 4 μm.

An effective quarter-wave plate should be able to transmit light waves of different polarizations with a high and approximately equivalent output amplitude over a wide spectral band. Further, the phase delay between the TE and TM modes should be kept constant at π/2 in the design frequency range. We therefore determine the conditions for achieving the constant phase delay of π/2 within the design frequency range for a double-stacked HMM waveguide array by incorporating the propagation characteristic of the HMM waveguides into the general transmission matrix (see Methods Section). When Eqs. (6 and 8) are satisfied simultaneously, the phase delay will be held at π/2 in the design frequency range. It has been previously demonstrated that the propagation constants for different polarizations are determined by the side dimension of the HMM waveguide^34^, so *k*_1_, *k*_2_ and Δ*k*_1_, Δ*k*_2_ as a function of frequency *f* can be uniquely retrieved. Thus, there exists only one pair of (H_1_, H_2_) that satisfies both Eqs. (6) and (8). If the actual height of the complete device deviates from the designed height, the introduced phase delay is no longer kept at π/2. In addition, it can be easily inferred from Eqs. (6 and 8) that the sign of *k*_1_, *k*_2_ (Δ*k*_1_, Δ*k*_2_) should be −1. When the side dimensions for the two HMM waveguide arrays are chosen to satisfy |*k*_1_| = |*k*_2_|, there exist no solutions to Eqs. (6 and 8). In other words, |*k*_1_| should be unequal to |*k*_2_|. When the side dimensions for the upper and lower HMM waveguides are designed such that the operation frequency approaches both cut-off frequencies for the TE (the lower HMM waveguide) and TM (the upper HMM waveguide) modes, both *k*_1_ and *k*_2_ are relatively large; thus, the resultant heights for the upper and lower HMM waveguide, H_1_ and H_2_, can be significantly reduced according to Eq. (6). Figure 3(a,b,c) show the dependence of *k*_1_ and *k*_2_, phase delay and transmission on the light frequency when the side dimensions are designed to produce relatively large *k*_1_ and *k*_2_. In the operation frequency range from 1 to 1.2 THz, the estimated Δ*k*_1_ and Δ*k*_2_ are −0.049 and 0.033 μm^−1^, and H_1_ and H_2_ are approximately 115 and 185 μm, respectively. It can be easily observed from Fig. 3(b) that the phase delay can be made around π/2 in the design frequency region. However, such a scheme results in low transmission [Fig. 3(c)], which can be attributed to the significant mismatch between the propagation constant of the HMM waveguide arrays and the wave number in air and to an increased absorption loss induced by the slow-light effect near the cut-off frequency. When the side dimensions for the upper and lower HMM waveguides are designed such that the operation frequency is far from each cut-off frequency for the TE (lower HMM waveguide) and TM (upper HMM waveguide) modes, both *k*_1_ and *k*_2_ are relatively small; thus, the resultant height of each HMM waveguide would be significantly increased according to Eq. (6). Figure 3(d,e,f) show the dependence of *k*_1_ and *k*_2_, phase delay and transmission on the light frequency when the side dimensions are designed to induce relatively small *k*_1_ and *k*_2_. In the operation frequency range from 1 to 1.2 THz, the estimated Δ*k*_1_ and Δ*k*_2_ are −0.009 and 0.003 μm^−1^, and H_1_ and H_2_ are approximately 400 and 1125 μm, respectively. Although the device benefits from the nearly dispersion-free phase delay of π/2 [Fig. 3(e)] and the relatively high transmission within the frequency range of interest [Fig. 3(f)], the overall device height is nearly five times that of the light wavelength.

**Figure 4:**
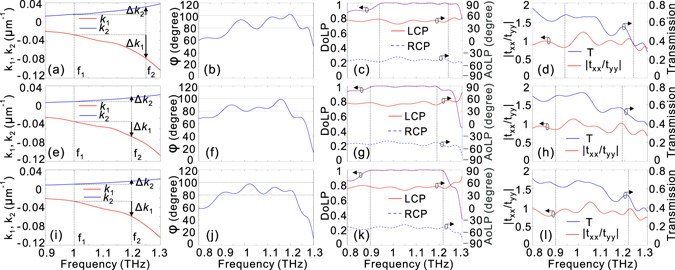
The performance of the three sets of double-stacked HMM waveguide arrays. *k*_1_ and *k*_2_ (**a**,**e**,**i**), phase delay (**b**,**f**,**j**), DoLP and AoLP with LCP and RCP input (**c**,**g**,**k**), and amplitude ratio of *t*_*xx*_/*t*_*yy*_ and total transmission T (**d**,**h**,**l**) as a function of *f* for the first (**a**–**d**), second (**e**–**h**), and third (**i**–**l**) double-stacked HMM waveguide arrays: (**a**–**d**) H_1_ = 85 μm, H_2_ = 255 μm, L_x1_ = 62 μm, L_y1_ = 18 μm, L_x2_ = 22 μm, and L_y2_ = 53 μm; (**e**–**h**) H_1_ = 95 μm, H_2_ = 380 μm, L_x1_ = 61 μm, L_y1_ = 17 μm, L_x2_ = 16 μm, and L_y2_ = 48 μm; (**i**–**l**) H_1_ = 80 μm, H_2_ = 400 μm, L_x1_ = 62 μm, L_y1_ = 20 μm, L_x2_ = 23 μm, and L_y2_ = 47 μm. The vertical dashed lines in (**c**,**d**), (**g**,**h**) and (**k**,**l**) represent the phase bandwidth of operation.

To effectively decrease the device height without sacrificing the transmission efficiency, the side dimensions need to be selected appropriately such that the operation frequency is away from the cut-off frequency. In this way, we can effectively avoid the high absorption from the HMM waveguides while still keeping the values of *k*_1_ and *k*_2_ at a relatively high level, which is beneficial for the miniaturization of the HMM wave plate. Figure 4(a,e,i) show the dependence of *k*_1_ (*k*_2_) on *f* for three sets of structural parameters for the upper and lower HMM waveguide arrays. It should be noted here that there exists some oscillation in the dispersion curves, which can be attributed to the Fabry-Perot effect caused by the HMM waveguides. In particular, since the propagation constant of the TM mode of the upper HMM waveguide is much larger than that of the TE mode of the lower HMM waveguide, the oscillation range of the *k*_1_ curves is bigger than that of the *k*_2_ curves. For the three sets of the double-stacked HMM waveguide arrays, −Δ*k*_1_/Δ*k*_2_ is approximately 3, 4, and 5 around 1.1 THz, respectively. By incorporating these values into Eqs. (6 and 8), we can obtain the heights of the HMM waveguides that enable phase delay of π/2 with the aim to convert linearly polarized light to left-circularly polarized light. It is interesting to see from Fig. 4(b,f,j) that by utilizing the three sets of structural parameters to form the double-stacked HMM waveguide arrays, the phase delay exhibits a nearly flat response within a wide spectral band around 1.1 THz. If the phase bandwidth is defined as the phase delay of 90° ± 10°^11, 14^, the corresponding phase bandwidths for the three sets of double-stacked HMM waveguide arrays are 0.94–1.24 THz, 0.9–1.19 THz, and 0.89–1.22 THz.

To quantitatively evaluate the performance of the quarter-wave plate by the double-stacked HMM waveguide arrays, we have calculated the DoLP and AoLP when a circularly polarized light wave propagates in the –z direction. DoLP is used to evaluate the degree of linear polarization of the transmitted wave, while AoLP describes the polarization angle of the linearly polarized light relative to the x-axis. DoLP and AoLP are defined as $DoLP=\sqrt{{s}_{1}^{2}+{s}_{2}^{2}}/{s}_{0}$ and *AoLP* = 0.5 tan^−1^(*s*_2_/*s*_1_), where *s*_0_, *s*_1_, and *s*_2_ are the Stokes parameters given by *s*_0_ = |*E*_*x*_|^2^ + |*E*_*y*_|^2^, *s*_1_ = |*E*_*x*_|^2^ − |*E*_*y*_|^2^, and ${s}_{2}={E}_{x}{E}_{y}^{\ast }+{E}_{x}^{\ast }{E}_{y}$, respectively^49^. Here, ‘*’ denotes the complex conjugate. It can be observed from Fig. 4(c,g,k) that the bandwidth over which the DoLP is nearly unity is basically consistent with the phase bandwidth shown in Fig. 4(b,f,j). More interestingly, the AoLP within the phase bandwidth where the DoLP is near unity is kept at approximately 45°. This insensitivity of AoLP to light frequency within the phase bandwidth originates from the weak dispersion in the amplitude transmission for different polarizations [Fig. 4(d,h,l)]. The weak frequency dependence of the AoLP has the advantage of fixing the fast and slow axes, which is highly desirable when implementing a quarter-wave plate in a practical situation. We have noted in recent studies on quarter-wave plates based on plasmonic metasurfaces that the AoLP changes significantly within the phase bandwidth of operation owing to the strong dispersion in the transmission/reflection coefficients^11, 17, 24, 26^. Figure 4(d,h,l) present the transmission spectra for the three sets of HMM quarter-wave plates, where linearly polarized light with the polarization angle of 45° relative to the x direction illuminates along the –z direction. It gets as high as 0.5 within the operation bandwidth, which is comparable to or even higher than that in Fig. 3(f). The reason that the transmission is limited can be explained as follows. First, there is a mismatch between the propagation constant of the HMM waveguide arrays and the wave number in air; hence, a portion of light will be reflected back into the air. Second, to effectively reduce the device height, an operation frequency band that is near the cut-off frequency has been selected for polarization manipulation, which comes at the cost of significantly increased absorption loss from the HMM waveguide arrays.

**Figure 5:**
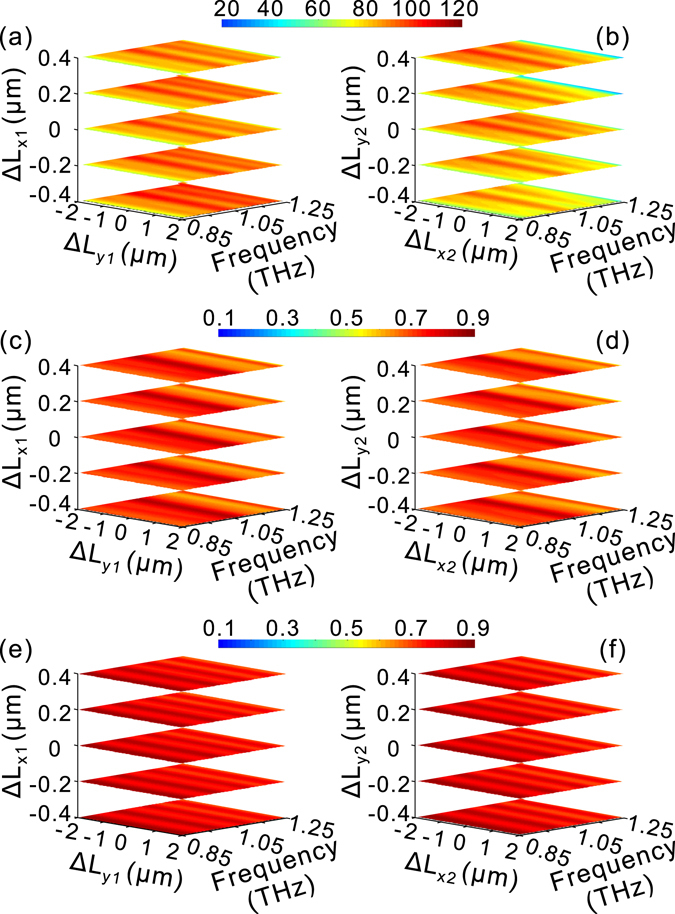
Influence of cross-sectional structural parameters on the performance of the third set of HMM quarter-wave plate in Fig. 4. The phase delay (**a**,**b**), amplitude of transmission coefficients for different polarizations, t_xx_ (**c**,**d**) and t_yy_ (**e**,**f**). In (**a**,**c**,**e**), ΔL_x1_ and ΔL_y1_ denote the variation with respect to L_x1_ (=62 μm) and L_y1_ (=20 μm), respectively, while L_x2_, L_y2_ are fixed at 23 μm and 47 μm, respectively. In (**b**,**d**,**f**), ΔL_x2_ and ΔL_y2_ denote the variation with respect to L_x2_ (=23 μm) and L_y2_ (=47 μm), respectively, while L_x1_, L_y1_ are fixed at 62 μm and 20 μm, respectively.

For an HMM waveguide, the mode propagation constant is highly dependent on the cross-section parameters. Therefore, the tolerance of the structural parameters of the HMM waveguide cross-section is very important for the design of HMM quarter-wave plates. Figure 5 shows the tolerance of the phase delay and transmission coefficients as a function of the cross-section parameters for the third set of the HMM quarter-wave plates. It can be observed that the phase delay is kept at around π/2 in the design frequency band even if the widths of the HMM waveguides change significantly [Fig. 5(a,b)]. Meanwhile, the corresponding amplitude transmission for different polarizations changes slightly in the frequency range of interest [Fig. 5(c–f)], which is beneficial for constructing a quarter-wave with high performance in terms of AoLP and DoLP. It is worth noting here that the variation in L_x1_ and L_y2_ has a much stronger influence on the phase delay than that of L_y1_ and L_x2_. This is because the TM (TE) mode for the upper (lower) HMM waveguide is closer to the cut-off frequency and hence more sensitive to L_x1_ (L_y2_).

**Figure 6:**
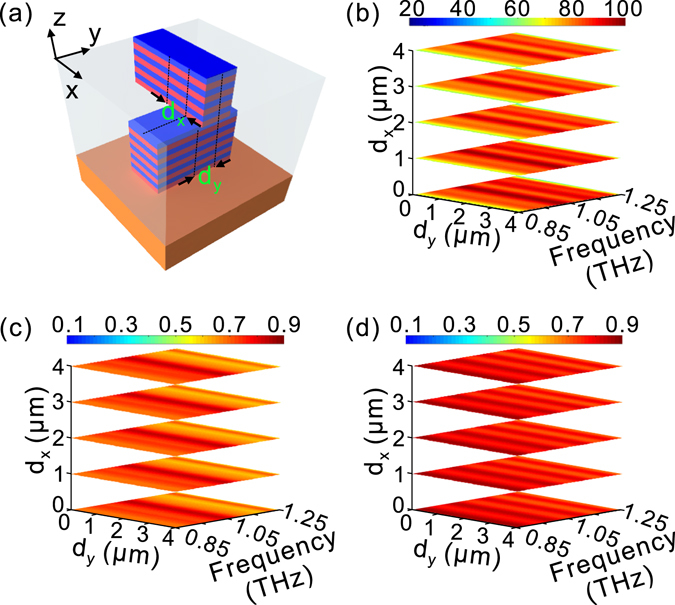
Influence of the dislocation on the performance of the third set of HMM quarter-wave plate in Fig. 4. (**a**) The sketch map of a unit cell of the double-stacked HMM waveguide arrays, in which d_x_ and d_y_ represent the dislocations between the upper and lower HMM waveguide arrays along +x and +y directions, respectively. The influence of misalignment on phase delay (**b**), amplitudes of transmission coefficients for different polarizations, t_xx_ (**c**) and t_yy_ (**d**).

We have also numerically studied the influence of misalignment between the upper and lower HMM waveguide arrays on the device performance. The dislocations along the +x and +y directions are denoted as d_x_ and d_y_ [Fig. 6(a)], respectively. The results show that the phase delay is slightly changed within the operation frequency range of 0.89–1.22 THz even if the misalignment is up to 4 μm [Fig. 6(b)]. Meanwhile, the amplitude transmission for different polarizations is accordingly kept high compared to the case without misalignment [Fig. 6(c,d)]. The small influence of misalignment on the device performance may be very suitable for fabricating a practical double-stacked HMM quarter-wave plate since it means that alignment between the upper and lower HMM waveguide arrays is not strictly required. We have also investigated the case of arranging a dielectric substrate for the upper HMM waveguide array so that the upper and lower HMM waveguide arrays are designed separately. The results demonstrate that the device performance can still be maintained (not shown here), indicating that the double-stacked HMM quarter-wave plate might be experimentally implemented by separately fabricating upper and lower HMM waveguide arrays. This may greatly facilitate the design and fabrication process in a practical situation.

## Conclusion

In conclusion, we have explored double-stacked HMM waveguide arrays to engineer the phase delay dispersion for broadband manipulation of light polarizations. The conditions for dispersion-free phase delay have been theoretically derived by incorporating the propagation characteristic of the HMM waveguide into the transmission matrix. The designed double-stacked HMM waveguide arrays are shown to be capable of maintaining the phase delay at around π/2 over a wide spectral band, which enables the conversion of linearly polarized light to circularly polarized light. Finally, the double-stacked HMM waveguide quarter-wave plates show excellent performance in terms of DoLP and AoLP, which may facilitate the implementation of a practical polarization converter in the terahertz domain. We emphasize that the presented design approach might be further extended to address the bandwidth issue for various metamaterials-based polarization manipulation devices^36–38, 50–52^.

## Methods

For the double-stacked HMM waveguide array [Fig. 2], the transmission matrix for describing the complex amplitudes of transmitted waves can be retrieved by taking into account the propagation characteristic of the HMM waveguide. Assuming a normal incident plane wave illuminates the double-stacked HMM waveguide arrays from the –z direction with the polarization angle θ relative to the x-axis, the electric field can be expressed as

1 $${{\bf{E}}}^{in}=[\begin{array}{c}{I}_{x}\\ {I}_{y}\end{array}]$$

where $$[\begin{array}{c}{I}_{x}\\ {I}_{y}\end{array}]=[\begin{array}{c}\cos \,\theta \\ \sin \,\theta \end{array}]$$ represents the Jones vector of the linear polarization. The transmitted electric field **E** ^*tr*^ can be written as

2 $${{\bf{E}}}^{tr}=[\begin{array}{c}{E}_{x}\\ {E}_{y}\end{array}]$$

where E_x_ and E_y_ denote the complex amplitudes of electric field along x and y directions, respectively.

The input and output electric field for the double-stacked HMM waveguide arrays can be related using the Jones matrix $${\bf{t}}=[\begin{array}{cc}{t}_{xx} & {t}_{xy}\\ {t}_{yx} & {t}_{yy}\end{array}]$$ as^53, 54^

3 $${{\bf{E}}}^{tr}={\bf{t}}{{\bf{E}}}^{in}$$

where t_ij_ (i, j = x, y) represents the transmission coefficient for i-polarized electric field with j-polarized electric field incidence. Similarly, for the upper and lower HMM waveguide arrays the Jones matrices can be expressed as $${{\bf{t}}}_{1}=[\begin{array}{cc}\begin{array}{c}{t}_{xx1}\\ {t}_{yx1}\end{array} & \begin{array}{c}{t}_{xy1}\\ {t}_{yy1}\end{array}\end{array}]$$  and $${{\bf{t}}}_{2}=[\begin{array}{cc}\begin{array}{c}{t}_{xx2}\\ {t}_{yx2}\end{array} & \begin{array}{c}{t}_{xy2}\\ {t}_{yy2}\end{array}\end{array}]$$. Here ${t}_{xx1}=|{t}_{xx1}|{e}^{i{k}_{x1}{H}_{1}}$, ${t}_{yy1}=|{t}_{yy1}|{e}^{i{k}_{y1}{H}_{1}}$, ${t}_{xx2}=|{t}_{xx2}|{e}^{i{k}_{x2}{H}_{2}}$, ${t}_{yy2}=|{t}_{yy2}|{e}^{i{k}_{y2}{H}_{2}}$, and *t*_*xy*1_ (*t*_*xy*2_), *t*_*yx*1_ (*t*_*yx*2_) represent the transmission coefficients for x-polarized (y-polarized) electric field with y-polarized (x-polarized) light illumination for the upper (lower) HMM waveguide array, respectively. *k*_*x1*_ (*k*_*x2*_), *k*_*y1*_ (*k*_*y2*_) denote the real part of the wave vectors of x- and y-polarization for the upper (lower) HMM waveguide array, respectively. Since an HMM waveguide array doesn’t convert x-polarized (y-polarized) electric field to y-polarized (x-polarized) electric field, *t*_*xy*1_, *t*_*yx*1_, *t*_*xy﻿2﻿*_ and *t*_*yx*2_ should be equal to zero. Consequently, the Jones matrix **t** for the double-stacked HMM waveguide arrays can be rewritten as

4 $${\bf{t}}={{\bf{t}}}_{2}{{\bf{t}}}_{1}=[\begin{array}{cc}{t}_{xx2}{t}_{xx1} & 0\\ 0 & {t}_{yy2}{t}_{yy1}\end{array}]$$

Jones vectors for the left- and right-circularly polarized light wave (LCP and RCP) are $$\frac{1}{\sqrt{2}}[\begin{array}{c}1\\ j\end{array}],\frac{1}{\sqrt{2}}[\begin{array}{c}1\\ -j\end{array}]$$, respectively. Therefore, we can get the conditions for achieving linear to circular polarization conversion with the double-stacked HMM waveguide arrays by combining Eqs. (3 and 4)

5 $$\frac{|{t}_{xx}|}{|{t}_{yy}|}=\frac{\sin \,\theta }{\cos \,\theta }$$

6 $$({k}_{y1}-{k}_{x1}){H}_{1}+({k}_{y2}-{k}_{x2}){H}_{2}=m\,\frac{\pi }{2}.$$

Here m = 1 (−1) denotes LCP (RCP). It is worth noting here, an HMM waveguide array has the ability to transmit light waves of different polarizations with a high and approximately equivalent output amplitude over a wide spectral band provided light frequency is far from the cut-off frequency [Fig. 1(e,g)]. Therefore, according to Eq. (5) it is highly expected that the polarization angle θ can be constantly kept at π/4 over a wide frequency band. The left side of Eq. (6) is the summation of the phase delay caused by the upper and lower HMM waveguide arrays. For simplicity, we rewrite *φ*_1_ = *k*_1_ *H*_1_, *φ*_2_ = *k*_2_ *H*_2_, where *k*_1_ = *k*_*y*1_ − *k*_*x*1_, and *k*_2_ = *k*_*y*2_ − *k*_*x*2_.

To ensure that the phase delay shows a flat response, the derivative of left-side of Eq. (6) should be equal to zero in a wide spectral band of interest

7 $${H}_{1}\frac{\delta {k}_{1}}{\delta f}+{H}_{2}\frac{\delta {k}_{2}}{\delta f}=0$$

where $\frac{\delta {k}_{1}}{\delta f}$ and $\frac{\delta {k}_{2}}{\delta f}$ represent the derivatives of *k*_1_ and *k*_2_ with respect to *f*, respectively. Considering *k*_1_ and *k*_2_ keep a nearly linear change with respect to *f* within a limited frequency range from *f*_1_ to *f*_2_, H_1_ and H_2_ should satisfy the following condition

8 $$\frac{{H}_{1}}{{H}_{2}}=-\frac{{\rm{\Delta }}{k}_{2}}{{\rm{\Delta }}{k}_{1}}$$

where Δ*k*_1_ = *k*_1_(*f*_2_) − *k*_1_(*f*_1_), Δ*k*_2_ = *k*_2_(*f*_2_) − *k*_2_(*f*_1_). Once the structural parameters of the HMM waveguide cross-sections are given, *k*_1_ and *k*_2_ as a function of *f* will be determined. As a result, according to Eqs. (6 and 8) H_1_ and H_2_ can be uniquely retrieved.

In this work, the propagation characteristic of the HMM waveguide array is estimated by numerical simulations with Lumerical finite difference time domain solutions. Periodic boundary condition is employed in the x and y directions, and perfect matched layer absorption condition is applied in the z direction. 5 and 20 mesh grids have been used to represent the thicknesses of Al and BCB layers, respectively, and 160 mesh grids are set in the x and y directions. We have also conducted simulations with even finer grid sizes, and the resultant propagation characteristic of the HMM waveguide array is almost unchanged, indicating that the grid sizes applied are sufficiently enough to accurately retrieve the propagation characteristic of the presented HMM waveguide.
